# Management of Acute Coronary Syndrome in Elderly Patients: A Narrative Review through Decisional Crossroads

**DOI:** 10.3390/jcm13206034

**Published:** 2024-10-10

**Authors:** Roberto Verardi, Gianmarco Iannopollo, Giulia Casolari, Giampiero Nobile, Alessandro Capecchi, Matteo Bruno, Valerio Lanzilotti, Gianni Casella

**Affiliations:** 1 Ospedale Maggiore Carlo Alberto Pizzardi, Largo Nigrisoli 2, 40133 Bologna, Italy; gianmarco.iannopollo@ausl.bologna.it (G.I.); giampiero.nobile@ausl.bologna.it (G.N.); alessandro.capecchi@ausl.bologna.it (A.C.); matteo.bruno@ausl.bologna.it (M.B.); valerio.lanzilotti@ausl.bologna.it (V.L.); gianni.casella@ausl.bologna.it (G.C.); 2 Cardiovascular Institute, Azienda Ospedaliero-Universitaria di Ferrara, Via Aldo Moro 8, 44124 Cona, Italy

**Keywords:** acute myocardial infarction, ACS, NSTEMI, elderly, older, revascularization, type 2 myocardial infarction, myocardial injury

## Abstract

Diagnosis and treatment of acute coronary syndrome (ACS) pose particular challenges in elderly patients. When high troponin levels are detected, the distinction between non-ischemic myocardial injury (NIMI), type 1, and type 2 myocardial infarction (MI) is the necessary first step to guide further care. However, the assessment of signs of ischemia is hindered in older patients, and no simple clinical or laboratory tool proved useful in this discrimination task. Current evidence suggests a benefit of an invasive vs. conservative approach in terms of recurrence of MI, with no significant impact on mortality. In patients with multivessel disease in which the culprit lesion has been treated, a physiology-guided complete percutaneous revascularization significantly reduced major events. The management of ACS in elderly patients is an example of the actual need for a multimodal, thorough clinical approach, coupled with shared decision-making, in order to ensure the best treatment and avoid futility. Such a need will likely grow throughout the next decades, with the aging of the world population. In this narrative review, we address pivotal yet common questions arising in clinical practice while caring for elderly patients with ACS.

## 1. Introduction

Age is arguably one of the strongest determinants of cardiovascular risk [1]. Current projections estimate that by 2050, 25% of the population will be older than 65 years old in western countries [2]. Despite reports of a recent reduction in its incidence, myocardial infarction (MI) remains a major driver of mortality and morbidity in older patients [3,4,5]. Registry data show that 25–40% of patients suffering from acute MI are >75 years old [6,7]. Moreover, MI is the most frequent presentation of Coronary Artery Disease (CAD) in elderly patients [8,9].

Nonetheless, older adults have been notoriously underrepresented or excluded from the major randomized clinical trials (RCTs) which dictate contemporary practice [10]. As a result, the applicability of such RCT data to these patients is challenging, as they present with different cardiovascular profiles than younger subjects, from risk factors [11,12,13] to atherosclerosis composition [9,14,15]. Moreover, attempts by regulatory institutions to increase their inclusion in trials have not yet produced significant results [16].

While the process of aging is inevitable, its impact on different organs and on global performance status is highly heterogeneous. In fact, it is commonly agreed that clinical decisions should not be based only on age [17]. Current European and American guidelines on acute coronary syndrome (ACS) do not provide specific indications for elderly patients, but recommend an individualized decision-making based on a multimodal evaluation [18,19].

Multimorbidity is associated with an increased risk of long-term Major Adverse Cardiovascular Events (MACEs) and all-cause mortality [20,21]. Bleeding risk is another key point to assess; however, since aging is associated with significant increments of both ischemic and hemorrhagic risk [22], the choice of the best antithrombotic regimen and its duration can be problematic.

Notwithstanding, it is clear that a thorough assessment of geriatric state and frailty is a key step in the management of older patients suffering from ACS. This topic has been extensively evaluated in a recent state-of-the-art review of this journal [23] and will not be discussed in this issue.

In this paper, we address pivotal questions arising in common clinical practice while caring for elderly patients with ACS:

- Firstly, we focus on the interpretation of high troponin levels: from the diagnosis of MI vs. Non-Ischemic Myocardial Injury (NIMI) to the differentiation of atherothrombotic Type 1 MI (T1MI) from mismatch-related Type 2 MI (T2MI).
- We then address the evidence of invasive vs. conservative management and myocardial revascularization of culprit and non-culprit coronary lesions.
- Finally, we mention some specific issues of the medical treatment in this patient population.

## 2. Myocardial Infarction or Non-Ischemic Myocardial Injury?

MI can be defined as an acute myocardial injury caused by myocardial ischemia [24]. Acute myocardial injury is diagnosed through the rise or fall of troponin levels above the 99th upper reference limit (URL), detected with guideline-recommended 0/1 or 0/2 h protocols [18].

### 2.1. Troponin

The 99th URL has been established for each company-specific troponin assay, using datasets of healthy reference subjects. An elevated troponin is a fairly common finding in elderly hospitalized patients, with reported prevalence of almost 50% in those older than 90 years [25]. However, in the majority of cases such alterations are not caused by MI, but by NIMI [25,26,27,28,29]. As a result, the positive predictive value (PPV) of the guideline-recommended threshold for diagnosing MI is significantly reduced in elderly subjects [26]. 

To address this issue, alternative diagnostic options have been considered:

- The use of an age-adjusted cutoff was evaluated in a secondary analysis of the High-STEACS study with more than 45,000 patients. In patients ≥75 years, a modest improvement in specificity (82.6% versus 91.3%) and PPV (51.5% vs. 59.3%) was observed. However, this was coupled with a marked reduction in sensitivity when compared with the use of the guideline-recommended threshold (55.9% versus 81.6%) [26].
- The difference in relative and absolute troponin change at serial testing (troponin kinetics) has been identified as a potential discriminator between NIMI and MI. However, while patients with T1MI showed higher absolute and relative changes on serial sampling, T2MI and NIMI values were similar and overall discrimination was only marginally improved [30].

In summary, differential diagnosis between NIMI and MI in elderly patients is not possible with serial troponin testing alone but relies on the assessment of signs of myocardial ischemia in the specific clinical scenario.

### 2.2. Non-Invasive Evaluation of Myocardial Ischemia

#### 2.2.1. Symptoms

Symptom interpretation in this population is particularly challenging:

- On the one hand, ACS is an infrequent diagnosis in elderly patients presenting with chest pain. For example, in a nationwide study, only 3.7% of patients ≥80 years presenting with chest pain had an ACS [31].
- On the other hand, it has been extensively described that older patients with MI present more commonly with atypical symptoms such as dyspnea, fatigue or non-specific discomfort, while chest pain may be frequently absent [32,33,34].

Extensive troponin testing in patients with non-specific complaints can generate confusion in result interpretation. For example, a cohort study analyzed the outcomes of 412 patients in which troponin was dosed in the emergency department (ED) due to atypical symptoms such as weakness, dizziness or fatigue. Mean age was 79 years, and among patients with elevated troponin, only 6% had an ACS, only one patient underwent coronary angiography (CA), and no patient received revascularization [35].

Despite this, real-world data suggest that the majority of T2MI is diagnosed through the presence of symptoms associated with troponin elevation (“subjective T2MI”), and not through objective evidence of ischemia (“objective MI”) [36]. As patients with “objective T2MI” showed worse prognostic outcomes, similar to those with T1MI, it has been proposed that more emphasis should be placed on the use of objective features of myocardial ischemia [37].

#### 2.2.2. ECG

ECG is the cornerstone in the assessment of objective myocardial ischemia. Repolarization abnormalities or Q-waves are the most frequent alterations that suggest MI [18]. However, ECG abnormalities are more prevalent in older adults without acute MI, reaching peaks of over 80% in patients with >90 years [38,39]. Such findings include conduction abnormalities, arrhythmias, and left ventricular hypertrophy. As a result, the detection of QRS or ST segment alterations is less specific for diagnosing acute MI in elderly patients [40,41].

For example, in an 80-year-old patient admitted for atypical chest pain and in whom a mild troponin elevation was documented, if ECG showed asymmetric ST depression in the context of ventricular hypertrophy (in the absence of a precedent exam for confrontation), it is clear that some additional effort should be pursued in order to diagnose acute MI.

#### 2.2.3. Echocardiography

The next fundamental step is the use of echocardiography, as the presence of Wall Motion Abnormalities (WMAs) can suggest acute ischemia [18]. Unfortunately, similar considerations as those for ECG abnormalities remain valid, as the prevalence of WMAs increases with age in the general population [42,43]. While CAD remains the most likely cause of WMAs in this age group, alternative causes are also more frequent, such as conduction abnormalities [40], cardiac amyloidosis [44], Takotsubo syndrome [45] or hypertensive heart disease [46]. Moreover, in the absence of a prior comparative imaging study, even if CAD is indeed the cause of WMAs, distinguishing acute myocardial ischemia from a chronic outcome of previous events can be problematic. Speckle tracking echocardiography and myocardial work quantification—which integrates measurements of left ventricular strain and pressure in a load-independent assessment—are promising discriminating tools, even if not yet widely available and implemented [47].

#### 2.2.4. Cardiac Magnetic Resonance and Coronary Computed Tomography Angiography

Cardiac magnetic resonance (CMR) is one of the most accurate diagnostic tools available in clinical practice to characterize myocardial pathologies. By allowing differentiation between coronary and non-coronary patterns of myocardial damage, its use could help in the differential diagnosis between MI and NIMI, without the need for invasive procedures.

As an example, in a recent paper from Oxford, the execution of CMR before CA in patients with suspected non-ST-elevation myocardial infarction (NSTEMI) documented non-ischemic pathologies in 18% of cases and normal findings in 11% [48].

Moreover, in the context of ischemic cardiomyopathy, the assessment of myocardial viability can help to predict future recovery of the systolic function and orient therapeutic decisions [49].

However, difficulty in tolerating the exam and complying with breath-holding indications can impair its execution in older patients; more importantly, the limited availability, its cost, and time consumption hinder its widespread use in the large number of elderly patients with suspected ACS.

Finally, the use of coronary computed tomography angiography (CCTA) could allow the diagnosis of CAD, avoiding the risk and costs of invasive procedures like CA.

In the context of chest pain, CCTA allows a non-invasive distinction between ACS, aortic dissection, and pulmonary embolism (triple rule-out), and can assess the patency of previously placed coronary stents [50].

However, its diagnostic performance is reduced in older patients due to the high probability of pre-existing CAD, coronary calcium, and hindered positive predictive value [51]. As for CMR, local differences in availability and expertise actually hinder its widespread diffusion.

To sum up, no single clinical, laboratory or instrumental parameter has proved sufficient for the differential diagnosis between MI and NIMI in elderly patients. All available elements should be balanced to formulate the diagnosis on a case-to-case basis and combined with a multimodal assessment of comorbidities and performance status in order to guide further care.

## 3. Type 1 or Type 2 Myocardial Infarction?

### 3.1. T2MI Definition

Once the diagnosis of MI has been established, an even bigger challenge is the distinction between Type 1 and Type 2 subgroups. T2MI is defined as an imbalance between myocardial oxygen demand and supply, not related to atherothrombosis [52]. Nosologically, non-atherosclerotic causes of coronary occlusion, such as embolism, spasm or dissection have also been included in the definition of T2MI. However, multiple pragmatic revisions have proposed the unification of all causes of coronary occlusion [53,54].

**Figure 1:**
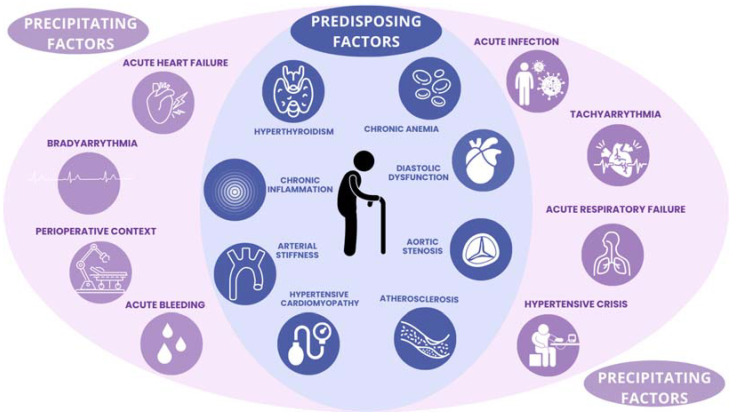
Predisposing and precipitating factors of type 2 myocardial infarction.

Nonetheless, excluding such cases, the oxygen supply/demand mismatch can be triggered by numerous acute cardiac and non-cardiac injuries (see Figure 1).

Unfortunately, such injuries are common in elderly inpatients and their presence does not exclude atherothrombosis. In fact, a rigid rule-out of T1MI in this subset of patients could cause an underestimation of its incidence, with consequent withholding of evidence-based treatments [55]. In this context, the troponin levels should be interpreted in light of the gravity of the identified trigger, considering the whole clinical picture. A disproportionate troponin elevation should prompt further investigation to exclude the diagnosis of T1MI.

Moreover, the documentation of coronary thrombosis is infrequent, even with the use of CA [9].

The differential diagnosis is also hindered by the heterogeneous definition of T2MI, as exemplified by the wide range of prevalence reported in previous studies [56,57]. In a retrospective analysis, when strict application of the fourth universal definition of MI was applied, misclassification of myocardial injury as T2MI was noticed in more than 40% of cases [58].

The uncertainty regarding this theme is so high that an international “Delphi method” study [59], with a pool involving 68 experts, reported that consensus on the topic of T2MI diagnosis was only achieved in 42% of the proposed statements [60].

One element of certainty is that the incidence of T2MI is strongly associated with increasing age, so much so that it has been defined as “an emerging geriatric disease” [61]. Such a definition is due to pathogenetic similarity with other geriatric conditions, where there is an interaction between the physiological aging process, predisposing chronic conditions (such as anemia or aortic stenosis), and acute precipitating factors [62]. Unfortunately, differential diagnosis becomes toughest when it is most needed, as the prevalence of T1MI also increases with age.

### 3.2. Multivariable and Biomarker Scores

In the attempt to simplify the diagnostic process, multiple strategies to discriminate type 1 from type 2 MI in the emergency department have been proposed.

While it is well known that T1MI patients have generally higher troponin levels, the quest for a specific troponin threshold to discriminate between T1MI and T2MI has been unsuccessful. For example, it was reported that a threshold as high as >50 times the URL would be required to achieve a PPV of only 75% for T1MI [30].

Neumann et al. proposed a multivariable score including female sex, chest pain characteristics, and TnI ≤ 40.8 ng/L. However, external validation in a large multicenter database showed a moderate discrimination (C-statistic: 0.67 (95% CI: 0.64–0.71)) [63,64].

Another potential strategy is the combined use of multiple biomarkers.

Natriuretic peptides such as N-terminal pro brain natriuretic peptides (NT-proBNPs) are a family of hormones secreted from the heart in response to pressure and volume overload [65]. It has been hypothesized that patients with T2MI may have higher cardiac wall stress, so the combined use of troponin and NT-ProBNP could help in the differential diagnosis. In a small cohort study, the NT-proBNP/Troponin T ratios were significantly higher in T2MI when compared to T1MI at baseline and 30, 60, and 180 min after presentation [66]. However, this strategy has not yet been tested in larger samples.

As infection is the most common trigger for T2MI, C reactive protein (CRP), a marker of acute inflammatory/infective state [67], has been evaluated as a potential diagnostic tool. In a cohort of 619 elderly patients with known CAD, Putot et al. showed that the CRP/troponin ratio had promising discriminating power, reporting a specificity of 90% in diagnosing T2MI versus T1MI when using a cutoff of 17.5 × 10^3^ [68].

More recently, the stress hormone copeptin has been proposed as a potential marker of T2MI in a small retrospective study involving 156 patients with elevated troponin and suspected MI. Patients with T2MI or myocardial injury showed significantly higher concentrations compared to T1MI [69]. However, the small size of the study does not allow specific conclusions.

Finally, multiple biomarkers of myocardial injury, endothelial/microvascular dysfunction, and hemodynamic stress have been cited as potential diagnostic tools. However, even the evaluation of 17 different molecules in a multicenter study with 1106 elderly patients did not provide a significant discrimination power for an early, non-invasive differential diagnosis [70].

In the future, the use of Machine Learning may have a role in guiding the differential diagnosis between NIMI, T1MI, and T2MI. However, recent attempts showed only a low overall diagnostic performance [71].

**Figure 2:**
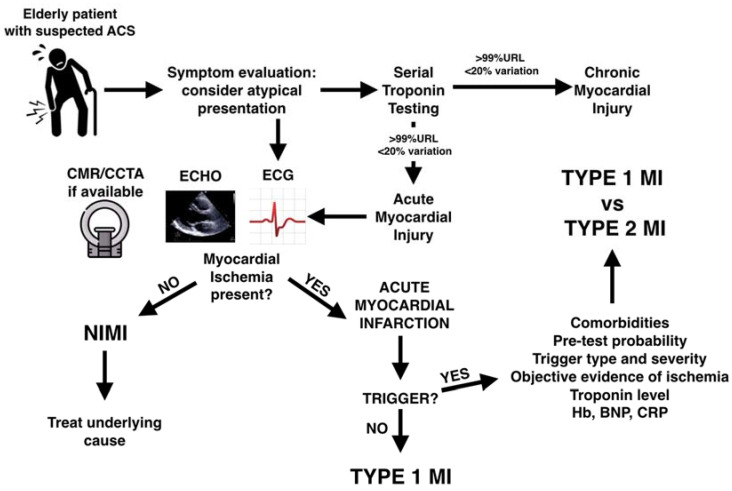
Diagnostic approach to elderly patients with suspected ACS. ACS = acute coronary syndrome; URL = upper reference limit; ECG = electrocardiogram; CMR = cardiac magnetic resonance; CCTA = coronary computed tomography angiography; MI = myocardial infarction; NIMI = non-ischemic myocardial injury; Hb = hemoglobin; BNP = brain natriuretic peptide; CRP = C reactive protein.

A possible diagnostic algorithm for elderly patients with suspected ACS is reported in Figure 2.

## 4. Invasive or Conservative Treatment?

In patients older than 75 years suffering from ST-elevation myocardial infarction (STEMI), the benefit of Primary Percutaneous Coronary Intervention (PPCI) over thrombolysis has been historically established [72]. More recently, such benefit was evaluated in a 20-year follow-up of the Zwolle study, at a time when all included patients had passed away. PPCI conferred a final survival gain of 1.5 years with a 29% increase in life expectancy [73]. In addition, registry data reported a significant benefit of PPCI even in nonagenarians [74,75].

On the other end, in elderly patients suffering from NSTEMI, the benefit of an invasive vs. conservative approach on hard outcomes is less evident.

Previous pooled subgroup analysis of historical RCTs of invasive vs. conservative management found that the reduction in MI recurrence at 5 years was mostly observed in older patients, while it was attenuated in patients aged <65 years [76].

Since 2008, six small RCTs conducted in five countries with 1479 subjects addressed this issue, and have been evaluated in a recent patient-level meta-analysis [77]. While all-cause and cardiovascular mortality were not different, the incidence of MI at 1 year was significantly lower in the invasive group compared with the conservative group (HR 0.62, 95% CI 0.44–0.87; *p* = 0.006). A limitation of this analysis was the relatively short follow-up of 1 year.

However, more recent reports in an extended follow-up of two of these trials substantially confirmed such results at 5 years [78,79]:

- The After Eighty Study was, until recently, the largest RCT comparing an invasive vs. conservative approach in elderly patients with NSTEMI [80]. At 5.3 years, the invasive strategy was superior to the conservative strategy in the reduction in the composite endpoint of MI, urgent revascularization, stroke, and death, with a gain in event-free survival of 276 days. Such a result was secondary to a significant reduction in MI and urgent revascularization, while no effect was detectable on mortality.
- MOSCA FRAIL was a multicenter study of 167 NSTEMI patients with frailty (Clinical Frailty Scale score ≥ 4) and a mean age of 86 years [81]. The recent analysis of 5-year outcomes showed that a higher 1-year mortality in patients randomized to invasive treatment was followed by a later benefit.

Importantly, the SENIOR-RITA study was recently published [82]. This was the largest RCT comparing invasive vs. conservative strategy in patients aged 75 or older with NSTEMI, enrolling 1518 participants across 48 sites in the UK. After a median follow-up of 4.1 years, the trial revealed no significant difference in the primary outcome of cardiovascular death or non-fatal MI between the two strategies. However, the invasive group experienced fewer non-fatal MIs and subsequent revascularizations. Notably, the initial benefit of the invasive strategy diminished over time, with the primary outcome curves converging at 2.5 years.

Characteristics of the available RCTs on invasive vs. conservative treatment of NSTEMI in elderly patients are summarized in Table 1.

In summary, current evidence shows no significant differences in mortality between invasive and conservative approaches yet highlights the benefits of invasive strategies in lowering non-fatal MI, subsequent revascularizations, and hospitalizations. In this regard, the impact of an invasive strategy on the patient’s quality of life deserves future research.

## 5. Complete or Culprit-Only Revascularization after STEMI?

Based on the available randomized clinical trials (RCTs), complete revascularization is recommended for patients with STEMI and multivessel disease [87,88,89,90,91].

However, some important nuances should be considered. The median age of the previous trials was around 60 years, and patients had limited comorbidities. Significant reductions in MACEs were observed only in the largest trial, the COMPLETE study, which had the added benefit of a reduction in coronary revascularization compared to the other RCTs.

Furthermore, the recent FULL REVASC trial showed no clear benefit of a complete revascularization vs. a culprit-only strategy in a general STEMI population, with longer follow-up (median 4.8 years) [92].

The EARTH-STEMI is a recent meta-analysis focused on data regarding cardiovascular death or MI over an extended follow-up [93]. This meta-analysis evaluated individual patient-level data from seven RCTs, including 1733 STEMI patients aged 75 or older with multivessel disease, who were randomized to either culprit-only or complete revascularization. Complete revascularization significantly reduced the composite endpoint of death, re-infarction, or revascularization within the first four years, though the difference between groups diminished over time. The reduction in the composite of cardiovascular death or re-infarction persisted throughout the entire follow-up period. However, long-term mortality rates were similar between the complete and culprit-only revascularization groups.

## 6. Complete or Culprit-Only Revascularization after NSTEMI?

While many trials evaluated complete vs. culprit-only percutaneous revascularization after STEMI, data on NSTEMI patients have been far scarcer.

The identification of the culprit lesion in NSTEMI is not always straightforward, especially in older patients who present more often with complex, multivessel disease. It has been reported that in more than 10% of patients with NSTEMI [94] a culprit lesion cannot be defined. Also, even when a culprit lesion is identified, the risk of misclassification is consistent. For example, an elegant CMR study reported a 31% rate of infarct-related artery misclassification [95]. In such cases, the use of intracoronary imaging can be considered in order to minimize the risk of culprit lesion undertreatment.

Also, the use of OCT in this context may help in the identification of T1MI subtype (plaque erosion vs. plaque rupture). In case of plaque erosion, stent placement may be safely avoided [96].

Until 2023, only observational studies on complete vs. culprit-only revascularization in the setting of NSTEMI had been conducted, with conflicting results. Even in such real-world studies, the representation of older subjects was low [97]. Nonetheless, elderly patients with NSTEMI and multivessel disease are largely undertreated with complete revascularization [98].

The FIRE trial was the first RCT to investigate complete vs. culprit-only revascularization after MI in elderly patients. The rationale of the proposed physiology-guided approach was to maximize the benefit of PCI for prognostic lesions, avoiding potential complications of unnecessary invasive treatment in physiologically “innocent” stenosis.

Patients older than 75 years who had undergone Percutaneous Coronary Intervention (PCI) of the culprit artery in the context of STEMI (35%) or NSTEMI (65%) were randomized to culprit-only or physiology-guided complete revascularization [99]. Multiple wire or angiography-based tools were allowed to assess the hemodynamic significance of luminal stenosis of non-culprit lesions. After 1 year, patients treated with complete revascularization had a significant 36% reduction in the composite outcome of cardiovascular death or MI. The incidence of adverse events such as contrast-associated acute kidney injury, stroke, or bleeding was comparable.

Recently, a subgroup analysis of the FIRE trial confirmed that in the NSTEMI subgroup, the use of a physiology-guided complete revascularization, compared with a culprit-only revascularization, significantly reduced the composite endpoint of death, MI, stroke, or revascularization at 1 year [100].

The FIRE and the SENIOR-RITA studies are arguably the most significant RCTs evaluating the benefit of PCI in elderly patients with NSTEMI. Since the designs of such studies differ significantly, comparisons are challenging. Nonetheless, we believe that the apparently conflicting results of such important trials deserve some comments:

1. The risk profiles showed notable differences, as the SENIOR-RITA population consisted of frailer patients, with higher rates of comorbidities, cognitive impairments, a larger proportion of women, and lower GRACE scores compared to the FIRE-NSTEMI population.
2. The timing of revascularization varied as well. In SENIOR-RITA, revascularization occurred after 5 days, while in FIRE-NSTEMI, it took place within 1 day. It is well established that earlier invasive strategies are linked to lower mortality in NSTEMI.
3. Complete revascularization was achieved in 100% of patients in the experimental arm of FIRE-NSTEMI, compared to only 54% in SENIOR-RITA.
4. The follow-up periods also differed, with FIRE-NSTEMI having a 1-year follow-up, whereas SENIOR-RITA extended to 4 years.

While the overall benefit of an invasive treatment in elderly patients with NSTEMI remains dubious, the results of the FIRE trial indicate a clear clinical benefit of a physiology-guided complete revascularization in patients with a clear and already treated culprit lesion.

## 7. Medical Therapy

### 7.1. Dual AntiPlatelet Therapy

Since more than 20 years, Dual AntiPlatelet Therapy (DAPT) has continued to be a cornerstone of the medical treatment of ACS. However, the discussion on its optimal duration has continuously updated prescription patterns. Older patients present both higher ischemic and bleeding risk. However, while ischemic risk decreases substantially and gradually after the first 1–3 months, bleeding risk remains substantially stable [101]. Moreover, previous attempts to prolong DAPT in older patients resulted in a net increase in the risk of bleeding, without a significant reduction in ischemic events [102].

European guidelines recommend a default 12-month duration after NSTEMI. However, they also state that in patients “not at high ischemic risk”, a shorter DAPT of 3–6 months should be considered (class of recommendation IIa). High ischemic risk can be defined as 3 vessels treated, ≥3 stents implanted, ≥3 lesions treated, bifurcation with 2 stents implanted, total stent length >60 mm, or chronic total occlusion [103]. 

In older patients, the net clinical benefit of a shorter DAPT was shown in a recent network meta-analysis, in which a reduction in major bleeding was observed in older adults for 1- and 3-month regimens when compared to longer regimens [104]. Given this evidence, shorter DAPT regimens should be the default strategy in elderly patients, reserving longer durations for specific cases of high ischemic risk.

### 7.2. Lipid-Lowering Therapy

Lipid-Lowering Therapy (LLT) is another fundamental part of optimal medical therapy after MI. Its use resulted in a significant reduction in MACE in multiple RCTs and is recommended for all patients by current guidelines [105].

This recommendation is based on the results of a 2019 meta-analysis of 28 randomized controlled trials that examined the reduction in MACEs in people older than 55 years on statin therapy [98]. In people aged 75 or more, statin therapy or a more intensive statin regimen was associated with a significant 21% reduction in MACEs per 1 mmol/L (39 mg/dL) reduction in LDL cholesterol [106]. 

More recently, an observational cohort in an older adult population (mean age 84 years) reported an association between a high-intensity LLT at discharge after MI and a reduction in all-cause mortality at 5 years [107].

However, side effects of statin therapy are more frequent in older patients [108], and special attention should be given to this age group, especially in the early post-discharge follow-up.

### 7.3. Blood Transfusion in Anemic Patients

Anemia is a frequent finding in elderly patients hospitalized for MI [109]. Whether to transfuse Red Blood Cell (RBC) Units in such patients is a common question faced by physicians. While on the one hand, blood transfusion may improve outcomes by optimizing oxygen delivery to ischemic myocardial cells, on the other hand, the risk of fluid overload, infection, and thrombosis may be deleterious. Current ESC guidelines do not provide any formal recommendation as to the optimal transfusion strategy and hemoglobin (Hb) target in anemic patients with MI [18]. Until recently, only small studies had been conducted, with conflicting results [110]. Observational data showed that the potential benefit of transfusion was largely dependent on hemoglobin threshold and age. For example, in patients aged ≥80 years and hemoglobin <8 g/dL, transfusion was associated with a 50% reduction in 1-year mortality [111].

In 2023, the larger MINT trial was published [112]: 3504 patients admitted due to MI and with Hb level < 10 g/dL were randomized to a restrictive (Hb cutoff for transfusion 7/8 g/dL) or a liberal transfusion strategy (Hb cutoff for transfusion 10 g/dL). The mean age was 72 years. After 30 days, myocardial infarction or death occurred, respectively, in 16.9% of the restrictive strategy group and in 14.5% of the liberal strategy group, without reaching the conventional target of statistical significance (*p* = 0.07).

However, an interesting subsequent correspondence debate pointed out that, despite not being significant from a rigorous statistical standpoint, the results of the trial may be clinically significant. For example, a Bayesian reanalysis of the data suggested that the probability of harm with a restrictive transfusion strategy ranged from 99.8% to 90.8% [113].

As the authors of the trial replied, this evidence supports the use of a liberal transfusion strategy in anemic patients with MI [114].

### 7.4. Polypill

Lack of adherence to medical therapy after MI is associated with worse outcomes [113]. Moreover, it has been estimated that adherence to secondary prevention drugs can be as low as 50% [115].

The SECURE study was an RCT evaluating the use of a polypill (containing aspirin, ramipril, and atorvastatin) vs. usual care in 2499 elderly ACS patients (mean age 76 years) [116]. After 3 years, the polypill strategy resulted in significant reduction in the primary outcome of cardiovascular death, non-fatal type 1 myocardial infarction, non-fatal ischemic stroke, or urgent revascularization, driven by a 33% significant reduction in mortality. Adverse events were similar between groups. On this ground, current guidelines state that a polypill strategy should be considered after ACS [18]. We believe that this consideration may be particularly true in older patients, because they were the target population of the SECURE trial, and they tend to have an increased pill burden. However, such a polypill is not yet available in many European countries.

## 8. Conclusions

The diagnosis and treatment of ACS in elderly patients is not a straightforward deal. High-quality evidence is still scarce, even if interest in this subject is growing. The distinction between NIMI, type 1 MI, and type 2 MI is the necessary first step to orient further care. Despite numerous attempts, no simple clinical or laboratory tool proved useful in this discrimination task. Current evidence suggests the possible benefit of an invasive treatment in terms of the recurrence of MI, with no significant impact on mortality. In patients with multivessel disease in which the culprit lesion has been successfully treated, a physiology-guided complete percutaneous revascularization significantly reduced MACEs, including all-cause death. The management of ACS in elderly patients is an example of the actual need for multimodal thorough clinical and critical approach, united with an honest, shared decision-making with patients and families, in order to ensure the best care and avoid futility. Such a need will likely grow throughout the next decades, with the aging of the world population.

## Figures and Tables

**Table 1:** Characteristics of RCTs on invasive vs. conservative treatment of NSTEMI in elderly patients. DM2 = diabetes mellitus type 2. Hb = hemoglobin. CKD = chronic kidney disease. COPD: chronic obstructive pulmonary disease. GFR = glomerular filtration rate. PAD: peripheral artery disease. NA = not available. AHF = acute heart failure. MI = myocardial infarction. CV = cardiovascular. HR = hazard ratio. IRR = incidence rate ratio. CI: confidence interval. § Time from randomization. §§ Time from admission. * Frailty: Clinical frailty scale (CFS) 5–7.

Trial	Enrollment	Population	Total Participants	Age (Median/Mean)	Sex (Women)	Comorbidities	Time of Angiography	Primary Outcome	Secondary Outcomes
Italian Elderly ACS Savonitto et al., 2012 [83]	2008–2010 Italy	NSTEACS ≥75 years	313	82	50%	DM2: 36.4% COPD: NA CKD: 45% Hb: 13.15 g/dL Prior Stroke: 7.9% PAD: NA Frailty: NA	1 day ^§^	Composite of all-cause mortality, non-fatal MI, CV rehospitalization for CV causes, disabling stroke, severe bleeding at 12 months: 27.9% invasive group vs. 34.6% conservative group; *p* = 0.26	MI: 11% invasive group vs. 17% conservative group; *p* = 0.27
After Eighty Tegn et al., 2016 [80]	2010–2014 Norway	NSTEACS ≥80 years	457	85	51%	DM2: 17% COPD: 9% CKD: NA Hb: NA Prior Stroke: NA PAD: 10.5% Frailty: NA	3 days ^§§^	Composite of MI, urgent revascularization, stroke, and death at median 1.53 years: 41% invasive group vs. 61% conservative group; *p* = 0.0001	MI: 17% invasive group vs. 30% conservative group; *p* = 0.001. Need for urgent revascularization: 2% invasive group vs. 11% conservative group; *p* = 0.001. Death from any cause: 25% invasive group vs. 27% conservative group; *p* = 0.53.
MOSCA Sanchis et al., 2016 [84]	2012–2014 Spain	NSTEMI ≥70 years	106	82	47%	DM2: 46% COPD: 31% CKD: 61% HB < 11 g/dL: 50% Prior stroke: NA PAD: 42% Frailty: NA	NA	Composite of all-cause mortality, recurrent MI, and readmission for revascularization or AHF at median 2.5 years: 67 patients in the invasive group vs. 56 patients in the conservative group; *p* = 0.877	All-cause mortality: 42% invasive group vs. 48% conservative group (95% CI 0.387–1.225)
80 + Study Hirlekar et al., 2020 [85]	2009–2017 Sweden	NSTEACS ≥80 years	186	84	42%	DM2: 19.3% COPD: NA CKD: 69% Hb: NA Prior Stroke: 13% PAD: 4,8% Frailty *: 15%	NA	Composite of MI, urgent revascularization, stroke, all-cause mortality, and recurrent hospitalization due to AF or HF at 12 months: 34.4% in invasive group vs. 37.4% in conservative group; *p* = 0.66	All-cause mortality: 11% in invasive group vs. 15.2% in conservative group, *p* = 0.40. Death and/or myocardial infarction at 12 months: 22.2% in invasive group vs. 28.9% in conservative group, *p* = 0.31.
RINCAL De Belder et al., 2021 [86]	2014–2018 United Kingdom	NSTEMI ≥80 years	250	85	47%	DM2: 20.9% COPD: 12.5% GFR: NA Hb: NA Prior Stroke: NA PAD: 3.2% Frailty: NA	Mean 2 days ^§^	Composite of non-fatal MI and all-cause mortality at 12 months: 18.5% invasive group vs. 22.2% conservative group; *p* = 0.39	No angina at 3 months: 85.9% invasive group vs 66.4% conservative group; *p* = 0.001. No angina at 12 months: 78% invasive group vs. 71.3% conservative group; *p* = 0.25
MOSCA FRAIL Sanchis et al., 2023 [81]	2017–2021 Spain	NSTEMI ≥70 years	167	86	53%	DM2: 55.6% COPD: NA Creatinine (mean): 1.35 mg/dL Hb: 12.4 mg/dL Prior Stroke: 17.9% PAD: 11% Frailty: mean value CFS 5.1	NA	Number of days alive and out of hospital: 284 days invasive group vs. 312 days conservative group; *p* = 0.12	Readmission due to all cardiac causes: 45.3% invasive group vs. 38.2% conservative group. Readmission due to bleeding: 8.9% invasive group vs. 2.9% conservative group (95% CI, 1.7–129; *p* = 0.02).
SENIOR RITA Kunadian et al., 2024 [82]	2016–2023 UK	NSTEMI ≥75 years	1518	82	45%	DM2: 30.6% COPD: 15.3% CKD: 20.7% Anemia: 50% Prior stroke: 15% PAD: 7.7% Frailty: median value CFS 3, FFIs 32% were frail	Median 2 days ^§^ 5 days ^§§^	CV death or non-fatal MI: 25.6% invasive group vs. 26.3% conservative group; *p* = 0.53.	Death from any cause or non-fatal MI: 42.4% invasive group vs. 42% conservative group; HR = 0.97; 95% CI 0.83– 1.13. Revascularization: 3.9% invasive group vs. 13.7% conservative group; HR = 0.26, 95% CI, 0.17–0.39.
